# A new method for endometrial dating using computerized virtual pathology

**DOI:** 10.1038/s41598-023-48481-y

**Published:** 2023-12-02

**Authors:** Yuval Or, Yoel Shufaro, Shlomo Mashiach, Bernard Czernobilsky, Sarit Aviel-Ronen, Liat Apel-Sarid, Mazal Dahan, Tsafrir S. Kolatt

**Affiliations:** 1 IVF unit, Kaplan Medical Center, 7642001 Rehovot, Israel; 2 Faculty of medicine, the Hebrew university, Jerusalem, Israel; 3 The Felsenstein Medical Research Center, the Sackler Faculty of Medicine, Tel-Aviv University, 69978 Tel-Aviv, Israel; 4 IVF unit, Rabin Medical Center, 4941492 Petach Tikva, Israel; 5 IVF unit, Assuta Medical Center, 69710 Tel-Aviv, Israel; 6 Patho-Lab Diagnostics Ltd., 7414001 Ness Ziona, Israel; 7 Adelson School of Medicine, Ariel University, 40700 Ariel, Israel; 8 Fertigo Medical Ltd., 3095303 Zichron Yaakov, Israel; 9 Iyar – The Israeli Institute for advanced research, 30900 Zichron Yaakov, Israel

**Keywords:** Outcomes research, Translational research

## Abstract

Endometrial dating (ED) is the process by which the menstrual cycle day is estimated and is an important tool for the evaluation of uterine status. To date, ED methods remain inaccurate and controversial. We demonstrate how the rise of computerized virtual histology changes the state of affairs and introduce a new ED method. We present the results of a clinical trial where magnified images of ex-vivo endometrial tissue samples were captured at different cycle days, together with measurements of serum hormone levels on the same day. Patient testimonies about their cycle day were also collected. Computerized image analysis, followed by statistical representation of the tissue features, allowed mathematical representation of the cycle day. The samples underwent ED histological assessment, which is currently the ED gold standard. We compared dating results from patient reports, serum hormone levels, and histology to establish their concordance level. We then compared histology-based ED with the new method ED in the secretory phase (i.e. post ovulation). The correlation coefficient between the two resulted in an R = 0.89 with a P-value of P < 10^–4^. The new method, Virtual Pathology Endometrial Dating (VPED), has the benefit of being a real time, in-vivo method that can be repeatedly applied without tissue damage, using a dedicated hysteroscope. One practical use of this method may be the determination of accurate real-time embryo transfer timing in IVF treatments.

## Introduction

During the menstrual cycle, the human endometrium follows a rapid structural and functional evolution in order to reach its optimal state for potential embryonic implantation. These changes occur under the control of the sexual steroid hormones and obey precisely defined series of metamorphoses until a receptive endometrium is developed. Endometrial dating (ED) is used to determine a point (state) of the endometrium along this time-dependent evolution line, e.g. the window of implantation (WOI).

Although variations in cycle length, stage phase and physiological expressions exist^1^, histology-based endometrial dating^2^, asserts that overall structure alteration is similar for different cycles of each woman and for different women. Histology-based endometrial dating has remained the gold standard^3^ to date but has been revisited and challenged many times since its introduction^4–6^.

Other endometrial dating methods, e.g. ultrasound imaging, were shown to have weak correlation with dating^7–9^. It remains controversial whether endometrial thickness alone or only the endometrial pattern shows correlation^10,11^. Sex hormone levels measured in the blood^12^ or urine, exhibit a well-defined pattern as a function of cycle progress^13^ and may thus serve as internal "clocks" for endometrial evolution, albeit indirectly.

Transcriptomic methods for dating^14^ estimates are based on the hypothesis that changes in the gene expression profile are tightly correlated with endometrial growth progression. Such methods are claimed to be superior in dating accuracy to Luteinizing Hormone (LH) peak dating. It seems that dating in general and endometrial dating in particular do not discriminate between women of fertile and infertile couples or evaluation of infertility^15,16^, yet some evidence indicates otherwise^15^.

The main motivation for the search for endometrial dating comes from its use to determine the WOI and precise timing for embryo transfer during in-vitro fertilization (IVF) treatments^4,17–22^. Accurate endometrial dating is expected to point to the true, biological (WOI)^23^. The WOI is usually defined as the period in the mid-luteal phase from (standard) day 19 to day 24, when implantation can take place. The WOI is time sensitive^22,24,25^ and usually occurs between (standard) days 19 and 21 of the cycle^26,27^. Yet, currently, the WOI is not well defined^8,13,28^ and variations in its timing are critical when synchronization is necessary during medical intervention. Therefore, endometrial dating plays an important role in targeting the accurate timing for embryo implantation. It is estimated that at least two thirds of implantation failures are due to insufficient uterine receptivity^17^ and thus, the need for a real-time precise determination of endometrial dating is crucial for IVF success^26,29–33^.

In this study we introduce and demonstrate a new method for endometrial dating that utilizes magnified images of the endometrial surface layer as means of “virtual-“ “pathology”, “histology” or “biopsy” to form and characterize statistical ensembles of tissue elements and attributes (VPED: virtual pathology endometrial dating).

We demonstrate the validity and limitations of this new endometrial dating method, using ex-vivo specimens of human endometrium. At a later stage the method will be tested in-vivo as well. We compare the new endometrial dating outcome measure, to the existing gold standard – the histopathological examination, as well as other methods for dating such as: patient’s menstrual diary, serum hormones level and sonography. We conclude with an estimation of the current accuracy of the proposed method and consider future avenues to maximize its utility and predictive power.

## Results

A total of 49 endometrial tissue samples were collected from 37 Recurrent Implantation Failure (RIF) participants in two medical centers. All 37 participating patients were in good health, aged 26 to 45 years. Twenty-eight patients had regular range (25–35 days) menstrual cycles. The remaining 9 reported having variances in the duration that were out of this regular range. Thirty of the participants had at least one previous pregnancy and twenty-six previously had at least one delivery. Table 1 summarizes the clinical characteristics of the trial population.

**Table 1:** Clinical characteristics of the trial population.

Cliniclal/demographic variable	Value (SE)
Population size	37
Sample size	49
Mean age in years	36.5 (4.6)
Median age (years)	37
Previous pregnancies	2.4 (2.7)
Previous deliveries	1 (0.85)
BMI (kg/m^2^)	24.1 (4.5)
Mean of average cycle duration (days)	28.0 (2.5)

**Figure 1:**
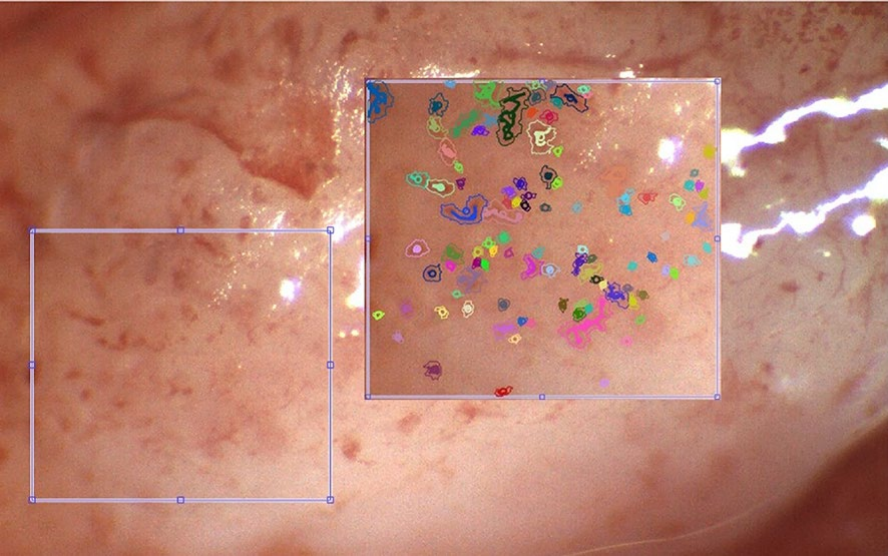
An example of a 4X magnification image of ex-vivo endometrial surface. The region of interest on the left (blue square, side ~ 800μm) is shown in the tissue context and then enlarged (on the right) along with overlaid computer- identified tisue elements.

A total of 344 images of the ex-vivo tissue samples were captured under 2X magnification and 625 images under 4X magnification. Figure 1 shows an example of an endometrial tissue image with its identified elements (computerized image analysis) overlaid. Patient reports about their menstrual cycle were collected and levels of four hormones [Estradiol, Progesterone, Luteinizing Hormone (LH) and Follicle Stimulating Hormone (FSH)] were analyzed from blood samples, collected on the same day of the tissue collection, and a transvaginal ultra-sound examination yielded endometrial thickness and appearance.

By patient reports, 37 specimens were collected after day 12 of the cycle, prior to any re-normalization to a standard 28-day cycle. Twelve participants underwent two endometrial scratching procedures during the same cycle: the first in the late proliferative phase and the second approximately 10 days later, during the secretory phase.

### Concordance level of traditional endometrial dating methods

Comparison of endometrial dating from the patient report and from a ${\chi }^{2}$ analysis of the hormone levels yields a correlation coefficient of $R=0.76$. The correlation coefficient of patient report and histological results yields $R=0.66$, with histology dating values systematically underestimating the cycle day in comparison to the patient report. Ultrasound-based endometrial thickness showed no statistically significant correlation with other dating methods.

### VPED–global tissue attributes

A computer vision algorithm identified pores (gland outlets) and blood vessels on images of the ex-vivo tissue originally facing the lumen and their various attributes (features such as diameter, shape, rim contour, etc.) were calculated. All images of the same specimen (~ 20 images per specimen in both magnifications) were collated to produce the entire list of identified tissue elements and their feature vectors. Global image attributes (e.g. pore density) were calculated for each image and averaged over all images. Probability Distribution Functions (PDFs) of each element feature were then calculated, referring to the tissue element population as a statistical ensemble.

The global tissue attributes (blood vessel density, pore number density and filling factor) did not show any statistically significant trend with cycle day as calculated by any one of the traditional dating methods.

### Comparison to traditional endometrial dating methods

**Figure 2:**
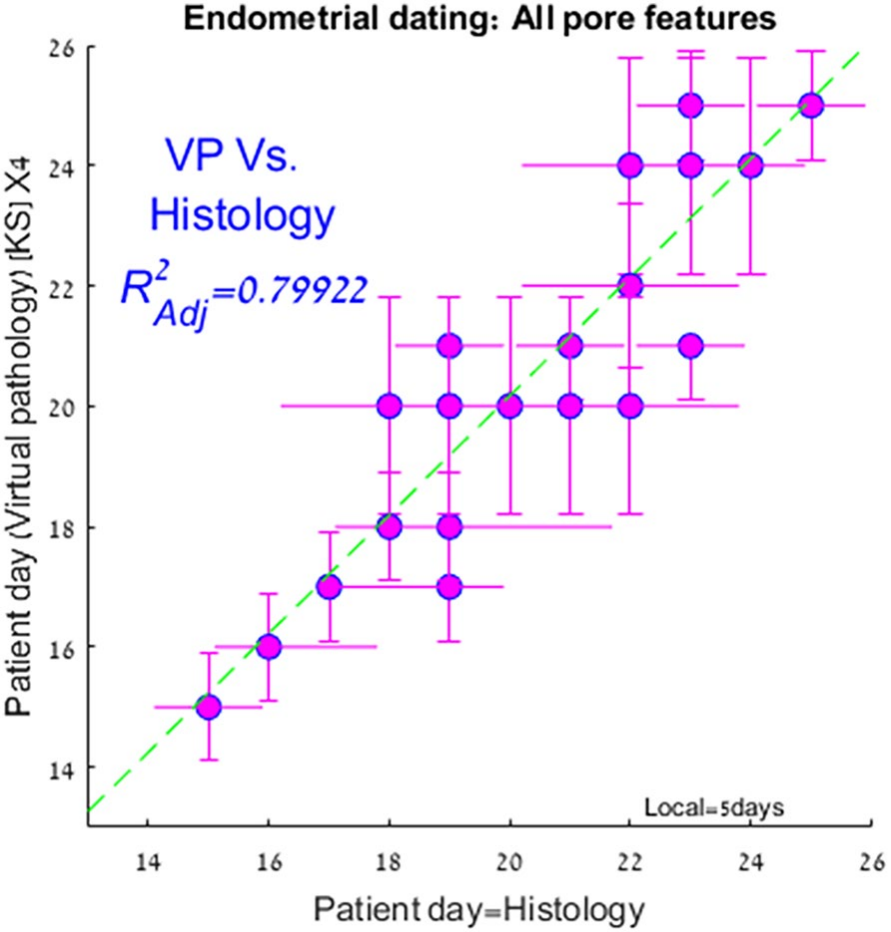
Comparison of virtual-pathology based endometrial dating with traditional, histology based dating. The virtual-pathology data are the averaged patient’s outcome, using all the pore features in equal weights for their KS-scores, convolved with a 5 day tophat window. The green line depicts the regression line yielding an adjusted correlation coefficient R = 0.89. Errors for the histology-based patient day are taken from the cluster–cluster comparison (distribution width) shown in Fig. 6. The derived P-value is < 8.4 × 10^–5^ (for the null hypotesis of no-correlation), however this P-value does not take into account systematic errors that may well exist within both estimates.

Figure 2 shows the concordance between the histology-based dating and the VPED from the analysis of the 4X magnification image set. Each patient’s (sample) pore-driven *PDF* was compared to the equivalent cycle day *PDF* via Kolmogorov–Smirnov (KS) statistics and the best matched (highest probability) cycle day is the indicated VPED inferred day (see Sect. “Image processing” under “Materials and methods” for details). We applied a five-day top-hat window to reflect the a-priory knowledge within the secretory phase. Errors for the VPED method (vertical) are calculated from the earlier derived cycle day accuracy KS test (cf. Fig. 6). The obtained correlation coefficient of 0.89 (R^2^ = 0.79) in the secretory phase, is very high, considering the built-in inaccuracies of the histology-based endometrial dating (horizontal errors). The nearly diagonal regression line, means there is virtually no demonstrable systematic bias in the method during this phase. Attempts to divide the samples into patients who previously gave birth and other patients, or were previously pregnant and the complementary group, did not yield any statistically significant difference between these groups regarding VPED and its correlation with other ED methods.

## Discussion

We demonstrated a novel method for endometrial dating. Even though the need for an accurate, real-time dating is evident, especially in the context of assisted reproductive technology, there is currently no gold standard. Histopathology remains the in-vitro gold standard but has been widely criticized. Endometrial thickness and gross structure, as obtained through ultra-sound imaging do not provide enough data for dating^10^. Hormones are measured in the serum and therefore provide only limited dating value for the target tissue^34^. Patient testimonies are partial, subjective and limited^35,36^. Transcriptomic methods may be relevant to some sub-population of IVF patients, but are in-vitro, not available in real-time and subject to controversies regarding their efficacy^37,38^.

To a very large extent, the initial and final conditions of the endometrial morphology for all cycles and women are identical. Indeed, an evolution course may exhibit a different pace at different phases, but it should go through all stages. The emergence of computerized vision, powerful computational capabilities and strong algorithms, have paved the way to follow these stages through virtual biopsies and image-based analysis and to provide accurate dating thereafter.

Using these advancements, we present a viable in-vivo method for the determination of endometrial dating that has currently been demonstrated on ex-vivo samples. Due to ethical considerations, the study population included RIF patients. These patients undergo endometrial biopsy collection one cycle prior to their IVF treatment cycle, as part of their preparation protocol^39^. We used this procedure to collect the analyzed samples. It is unclear whether this choice introduces inherent bias^40,41^. When image capture via hysteroscopic microscopy is used in-vivo, this limitation will be removed. At this stage of the method implementation, in the absence of a better alternative, we relied on the histopathology-based gold standard and correlated it with the VPED results. Comparisons of histology-based dating to other methods (hormones, testimonies) set the maximal accuracy for each one of the compared methods and sets the bar for histology dating variance (e.g. inaccuracy). On-going, in-vivo, data collection should gradually lead to higher accuracy of VPED until reaching the true, underlying, biological variance. If the variance in histology-based dating eventually turns out to be larger than the one in VPED, the present method will be able to determine this and may possibly replace the former and rid of the interobserver scatter^42–45^.

Morphology-based tissue characterization must resort to statistical measures (e.g. Ref.^46^). We therefore introduced a rigorous evaluation for such measures, and related it to previously and similarly attained results. For instance, lack of positive correlation of global image features (e.g. pore number density, pore filling factor) is consistent with previous findings (gland to stroma ratio) based on histology^6^. Murray et al.^6^ found that gland configuration was a better dating indicator concurring with our findings for the glands’ (i.e. pore) rim attributes.

We went beyond global features and found that *distribution functions* (PDFs) of tissue elements are a better way to represent and express the tissue nature. The statistical approach allows us to sketch the standard “trail” of the endometrium evolution and incarnate it in mathematical forms. A side benefit of this approach is the fact that digital information, and in particular the concomitant statistical measures, is much more comprehensive and repeatable, less subjective, and less open to interpretation with respect to histopathology. This approach also provides interesting information about the tissue *evolution*.

**Figure 3:**
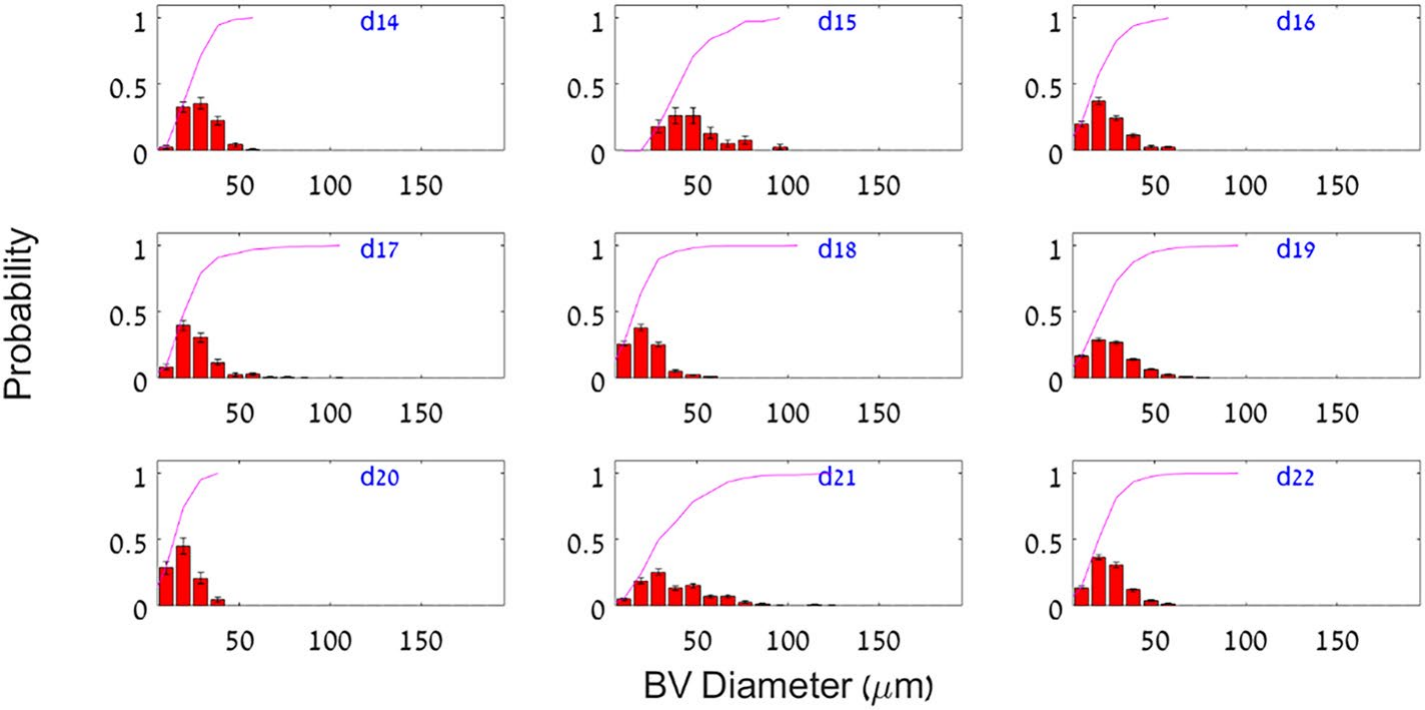
Measured probability distribution histograms of the blood vessel average diameter (4X magnification set) during the secretory phase. Each pane depicts the (weighted) average histogram of all patients that share the same cycle day (d# in blue, upper right corners) as determined by histology. Solid lines extend to the last histogram bin and describe the cummulative probability functions (cPDF_bv_) in each cycle day.

Figure 3 shows an exemplifying panel for such PDFs, in this case of blood vessel (BV) diameter *and* each histology-based *cycle day* (pane) from the 4X magnification image set, obtained from all patients that shared the same histology-based cycle day. The solid lines on top of each histogram are the integrated cumulative *PDF* (*cPDFs*) of each cycle day. Similar to the BV diameter PDFs in Fig. 3, *each feature of each tissue element* has its own time-dependent $PDF$ and the complementary, integrated $cPDF$. These *cPDFs* can be mathematically modeled and parametrized. We chose to use the Weibull function with two (relevant) parameters ($\gamma$, $\lambda$).

**Figure 4:**
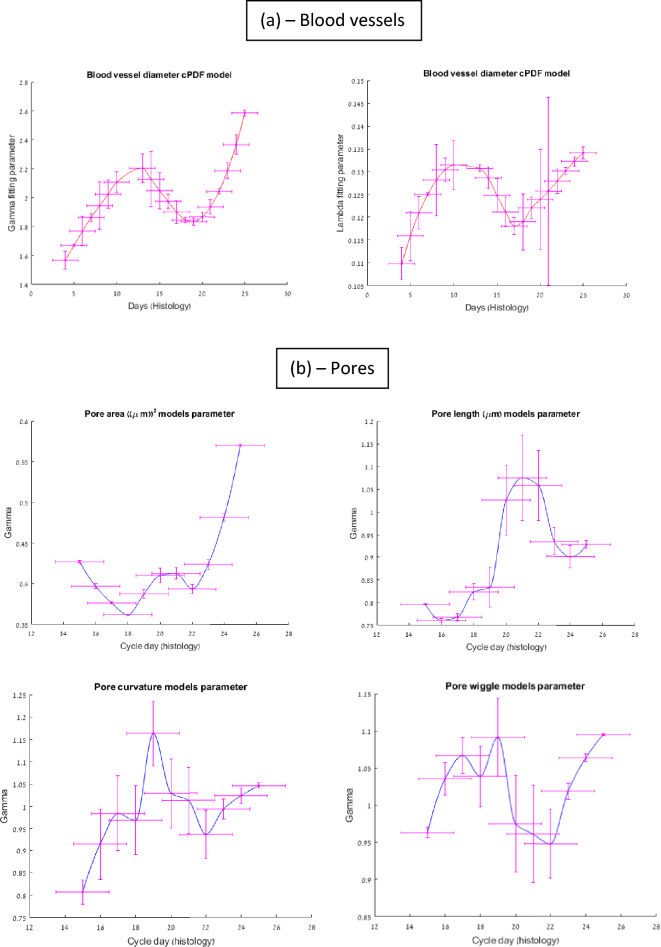
The average evolution of the Weibull function fitting parameter for cPDFs of blood vessels and pore features as function of the cycle day (histology-based 4X set) in the secretory phase. The depicted errors originate from finite sampling (vertical) and inaccuracies in histology ED evaluation (horizontal). (**a**) Blood Vessels: left—Gamma ($\gamma$) Right–Lambda ($\lambda$); (**b**) Pores: Upper left: pore area, right: pore length, lower left: pore’s rim curvature, right: pore rim’s wiggly (tortuosity) nature).

Turning to Fig. 4a which depicts the evolution of the BV cPDF parametrization, we notice that the $\gamma$ parameter of the distribution is always bigger than unity, meaning that there is a distribution maximum away from the resolution limit (the smallest BV diameter that can be detected). The parameter increases until day ~ 14, signifying the BV growth in diameter. However later on (days 13–19) this parameter starts to decline, probably due to the capillary angiogenesis next to the lumen, and the distribution peak veers to smaller values. Another turning point occurs near day 19, indicating that BVs become thicker again, probably to nourish these smaller ones that came into being. The right panel supports a consistent course of events of a distribution that first becomes skewed, then narrower and then skewed and stretched due to the additional large diameter BVs.

Examining the pore area parameters in Fig. 4b (upper left panel) we notice that $\gamma$ is always smaller than unity. That means the distribution (that is the PDF, *not* the cPDF) is inclined toward the smallest area pores throughout the endometrial evolution (during the secretory phase). This predominance of small (usually round) pores prevails until ~ day 18, when a new population of larger area pores starts to emerge, probably due to the mergering of existing pores. Combined pores contribute to the PDF tail leading to a more skewed PDF. If this was the only process, there would be a decrease in pore number density. As such a negligible decrease is observed, we assume that there must be a competing process of the emergence of small pores when gland outlets reach the lumen. While mergers dominate the competing two processes until day ~ 20–21, a substantial (relative) number of small pores pop up on days ~ 21–22. When this, as well as the older pore population, merge once again, the $\gamma$ value rises (days 22–25), but never exceeds unity.

The merging process is also reflected in the pore length distribution (Fig. 4b upper right). Since mergers will contribute to longer (usually elongated) pores, the $\gamma$ parameter of the PDF at the peak merging epoch (days 20–22) exceeds unity to have a PDF maximum, away from the resolution limit (abscissa “0”). On the lower left panel of Fig. 4b we notice how the curvature follows the same pattern: since small pores usually exhibit higher curvature we see the rise in $\gamma$ that precedes the strong merger episode and subsides beyond day ~ 19, to rise back only beyond day 22 but to a much smaller extent, as the typical pore size at that time is bigger. Similar trends can be observed in the lower right panel for the tortuosity parameter for similar reasons. The jagged rim of pores indicates their maturity and the merger events they experience during their lifetime. Taking all the above feature parametrizations together, enabled us to reach the high correlation presented in Fig. 2.

Despite the above limitations, the VPED predictions still match other endometrial dating methods with a high correlation coefficient of 0.89 when compared with the histological method. This is partly due to the fact that we used all the pore features simultaneously in order to calculate the most probable cycle day.

**Figure 5:**
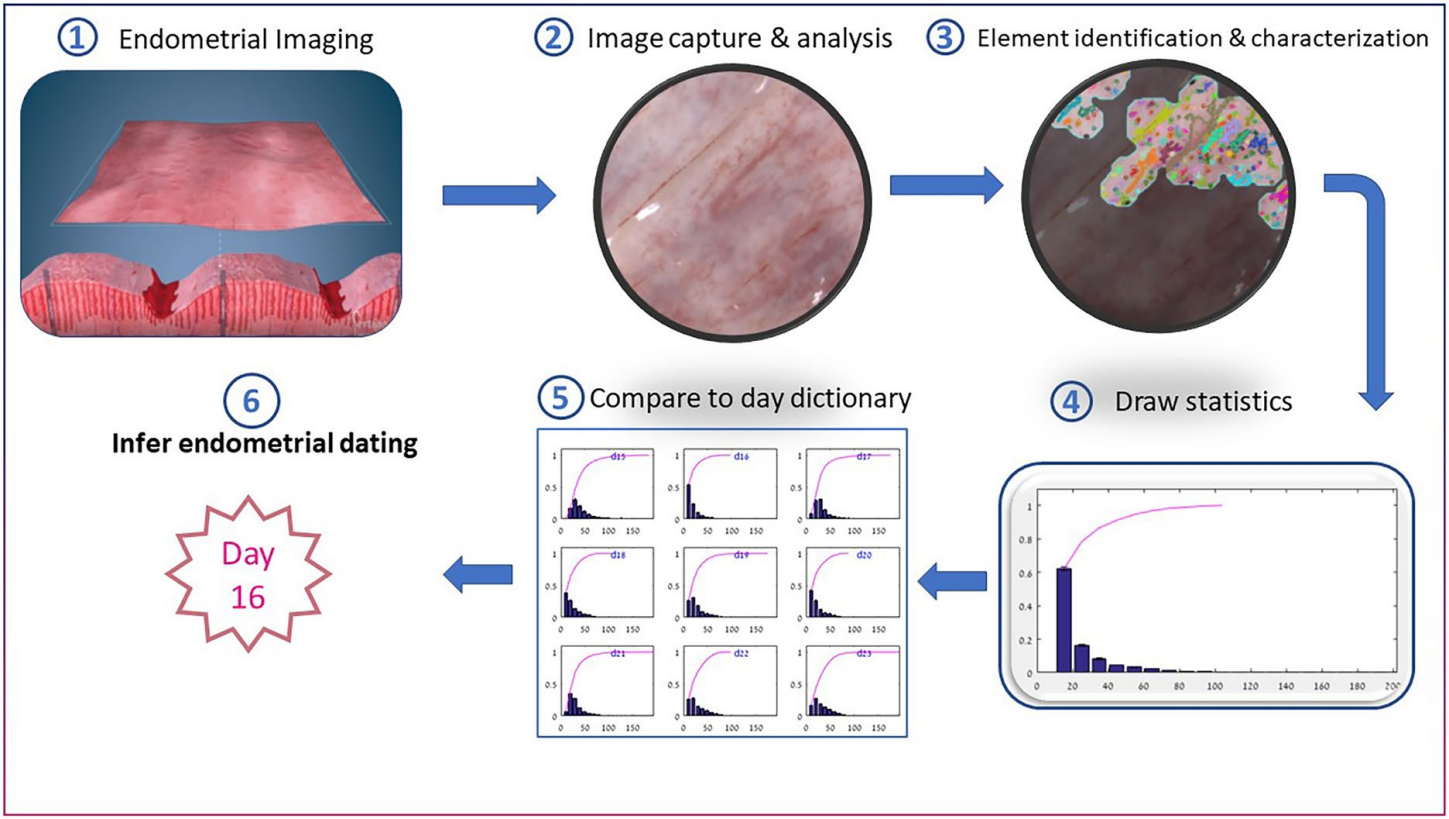
Endometrial dating method steps. (i) Get access to the endometrial surface facing the uterus lumen (ii) Capture a magnified image of the endometrium surface (iii) locate all image regions in focus and identify the relevant tissue elements (iv) calculate probability distribution functions for various element features (v) compare the obtained distributions with known cycle day distributions ("dictionary") (vi) deduce the most probable cycle day from the results of the previous step comparisons.

This study has established the VPED method for the first time, that in principle can be applied to in-vivo images through specially-designed hysteroscopic equipment and techniques. The VPED method may even replace the histology-based ED as a gold standard if proven to be more consistent and accurate. Figure 5 describes and summarizes the main steps of this new method.

Although we demonstrated the method using ex-vivo data, a significant advantage of an in-vivo VPED method will be the ability to image and evaluate the endometrium state more than once during the IVF treatment cycle and thus determine the best day for embryo transfer. This could be accomplished by morphologically monitoring the endometrium with a dedicated, mini-hysteroscope in the early secretory phase. The difference between the calendar cycle day and the VPED calculated cycle day is then translated into a slope. The slope indicates the pace of evolution of the particular endometrium during this particular cycle. This enables the prediction of whether and when the endometrium should reach its prime receptivity, and thus suggests the optimal ET timing in calendar days. If the asynchronization with the embryo age is significant, in a fresh IVF cycle, freezing the embryo for a later cycle is recommended. In a frozen/thawed cycle an earlier or delayed ET is recommended. Since a substantial share of IVF treatment failures are due to asynchronization, a relatively small investment in a 5-min procedure may be of particular clinical and economic benefit, saving unnecessary treatments, preventing healthy embryo wastage, reducing patient distress and reducing costs. A small fraction of a treatment cycle cost, may lead to a much higher live-birth rates in IVF and patient satisfaction.

## Materials and methods

### Study design

All methods were performed in accordance with relevant guidelines and regulations. We recruited IVF patients who were diagnosed with Recurrent Implantation Failure (RIF), namely at least three previous failures of IVF treatments. An endometrial biopsy was performed using *Pipelle* catheter (Pipelle ©CCD Mark II).

The key inclusion criteria for the participants were (i) IVF patients diagnosed with RIF who were regularly ovulating; (ii) age: 18–40; (iii) patients whose fertility status was unknown, or patients who were proven to be fertile (previous successful pregnancy).

Key exclusion criteria were (i) patients with known existing endometrial pathology; (ii) patients with a known history of infertility due to oligo-ovulation or anovulation; (iii) patients with a medical history of malignant tumors in their reproductive system; (iv) patients on any hormonal medications or treatments (excluding hormonal contraception in the previous cycle); (v) patients on hormonal contraception treatment in their current cycle; (vi) patients with an IUD; and (vii) Patients who were menstruating on the day of the biopsy collection. All participants provided written informed consent to take part in the study. The only recorded protocol deviations were with respect to the patient’s age upper limit (5 cases), due to shortage of patient availability during the Covid-19 pandemic. Yet, they had a normal cycle, and 4 of those 5 patients, had a previous successful pregnancy.

We focused mainly on the secretory phase for two reasons: (i) in future application we will determine the WOI in a real time in-vivo setting (ii) the gold standard histology-based endometrial dating is much more accurate in the secretory phase.

#### Endometrial dating calculation–traditional methods

For each participant the cycle dating was performed by the following four methods:

1. Patient report of the first day of her last menstrual cycle, her average cycle and menses duration and the variance in both. The *next* first day of menstruation, if available, was also documented.
2. Histopathological evaluation, commonly regarded as the gold standard method for cycle dating.
3. Blood sample for hormone level analysis.
4. Endometrial pattern and thickness as measured by ultrasound examination.

Prior to the biopsy collection procedure, each patient underwent a blood test and a transvaginal ultrasound (US) examination. Four hormone levels were measured: Progesterone (PR) and estradiol (ER) were analyzed through an Access 2 analyzer (Beckman & Coulter); luteinizing hormone (LH) and follicle-stimulating hormone (FSH) were analyzed using the Advia Centaur XP (Siemens). Measurement error estimates from each analyzer were included. The pivotal day (“day ‘0’ ”) within these hormone profiles is defined by the manufacturer relative to the LH-peak and the cycle duration is taken to be of 30 days^47^. The endometrial thickness was measured and documented during US examination.

### Histology

Following image acquisition, all samples were soaked in a 4% formaldehyde solution and delivered to a GLP certified histopathology lab (Patho-lab, Nes Ziona, Israel).

Sections of 4 µm were H&E stained and blindly analyzed, independently, by two expert gyneco-pathologists. Each pathologist completed a scaled questionnaire with common histology-base criteria^48,49^. Both experts had to come to consensus regarding the most probable cycle day. Any differences were recorded as part of the asymmetric error estimate of the diagnosis.

### Hormone levels

Cycle Day calculation by hormone levels in serum is performed using standard ${\chi }^{2}$ statistics including all four hormones along with their measurement errors and tabulated variance^47^.

### Cycle re-normalization

Whenever the duration of the cycle (patient report or blood sample model) was not of 28 days duration we re-normalized it to a 28-day period using a renormalization formula that re-scales the proliferative phase to 14 days. Note that histology-based dating is already normalized through its own reference calibration.

### Image processing

The fresh ex-vivo samples (< 3 h post collection) were examined under a stereoscope (Motic SMZ-171-TL) equipped with a ring 144-Led white light illumination source and a mounted camera (Motic MotiCam3). An observer captured images within the specimen regions of interest (ROIs), i.e. facing the uterus lumen (2X and 4X magnification).

### Statistical analysis 

Tissue characterization may be reduced to a single parameter or alternatively to functions. Single parameters calculated were (i) blood vessel density (ii) pore number density (i.e. number of pores in unit area) (iii) pore filling factor (i.e. the tissue fraction covered by pore area). Pore prominent features include (i) area (or radius of an equivalent circular disc) (ii) length (to signify deviation from circularity) (iii) rim curvature (iv) rim tortuosity ("wiggles"). The prominent blood vessel features are their diameter and length between splits.

Probability Distribution Function (PDF) was calculated for each feature and each sample (all images of the sample, 2X magnification and 4X magnification sets separately) (hereafter: "sample PDF"). In addition, PDFs for each feature were derived from all samples that shared the same cycle day according to the histology-based dating (hereafter: "cycle day PDF").

Each PDF translates to cumulative PDF (cPDF) and was mathematically modeled by a functional model.

#### Ensemble probability distribution functions

The $cPDF$ functions are modeled here by the three-parameter Weibull function.

$$We\left(x\right)=1-exp\left(-{\left(\frac{x-b}{\lambda }\right)}^{\gamma }\right).$$

The horizontal shift, $b,$ ("location parameter") is dictated by both the biology (e.g. smallest tissue element possible) and the spatial resolution limit for this specific test, and the magnification level. The "shape parameter", $\gamma$, depicts the slope, namely the pace at which the function reaches unity (in log–log space). The "scale parameter", $\lambda$, is related to the total width of the distribution, namely the range it spans. Basically, for $\gamma <1$, (at constant $\lambda$ ) the *PDF* (*not* the *cPDF*) is heavily skewed towards the left side of the histogram where the maximum is obtained (at the lowest resolution limit, similar in shape to $y=1/x$). For $\gamma >1$, the *PDF* reaches a maximum at a value higher than the resolution limit and as $\gamma$ increases, the PDF become narrower on both sides of the maximum. The $\lambda$ parameter stretches the distribution of the PDF and makes it more skewed. Please turn to the discussion section for the meaning of these parameters in the current context.

Figure 4a shows the average (over neighboring days) evolution of the $\gamma$ and $\lambda$ (Gamma and Lambda, "shape" and "scale" parameters respectively) of the Weibull function as calculated from the *cPDFs* of the average BV diameter throughout the cycle days. Figure 4b depicts the same for the γ parameter used in the modelled *cPDF* for the *pore features* focusing on the secretory phase alone. Data shown are for the 4X magnification set as dated by histology.

#### Comparisons of ensemble probability distribution functions

**Figure 6:**
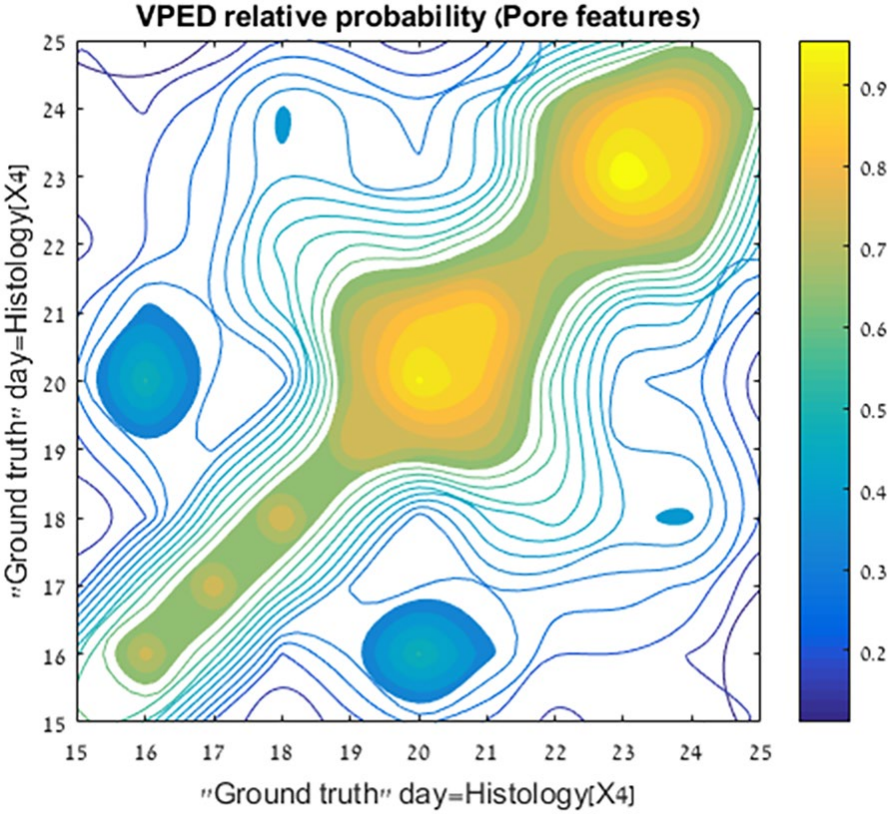
Relative probability of assessing the “ground truth” endometrial dating. Ground truth is taken to be histology. Patients of the same day (as determined by histology) are collated together to form the statistical distributions of four pore features for that cycle day. PDFs of different cycle days are then compared via KS-test (see text.) The KS values are convolved with a 3X3 Sobel kernel to reduce local noise and obtain the relative probability. The tight diagonal shape signifies the steep descent of probability for wrong day determination. Variances between patients who shre the same histology-based cycle day, translate to wider probability at these cycle days.These variances all remain within the ± 1 day limit. This broadening can be fully ascribed to inaccuracies in the histology-based endometrial dating.

Comparison of *cPDFs* between different cycle days provides quantitative way to assess the accuracy of the cycle day sample models. Each pair of *cycle day cPDFs* serve as an input to a Kolmogorov–Smirnov test (two-samples, KS). The KS test is meant to exclude resemblance of *PDFs* for two unrelated distributions. Figure 6 shows the relative probability distribution (smoothed with a [3 × 3] 2D Gaussian kernel) for each pair of cycle day *cPDFs* (4X set). The resemblance or strangeness of each pair (day $i$ versus day $j$) is shown relatively to maximal resemblance (the diagonal). The resultant probabilities, as they appear on the plot, provide the error estimate for the next stage, where individual *sample* PDFs are compared to the "underlying truth", namely all of the *cycle day PDFs*.

### Ethical approval

The study was approved by Kaplan Medical Center Ethics Committee (# 0193-17-KMC) and the Rabin Medical Center Ethics Committee (# 0399-19-RMC). It has been registered under the NIH clinical trials registry (ID NCT04288843).

## Data Availability

The data that support the findings of this study are available from Fertigo Medical Ltd. but restrictions apply to the availability of these data, which were used under license for the current study, and so are not publicly available. Data are however available from the authors upon reasonable request from Dr. Yuval Or, or Dr. Tsafrir Kolatt, and with permission of Fertigo Medical Ltd.
